# Targeted Mitochondrial Therapy With Over-Expressed MAVS Protein From Mesenchymal Stem Cells: A New Therapeutic Approach for COVID-19

**DOI:** 10.3389/fcell.2021.695362

**Published:** 2021-06-11

**Authors:** Amirhesam Babajani, Pooya Hosseini-Monfared, Samin Abbaspour, Elham Jamshidi, Hassan Niknejad

**Affiliations:** ^1^Department of Pharmacology, School of Medicine, Shahid Beheshti University of Medical Sciences, Tehran, Iran; ^2^Functional Neurosurgery Research Center, Shohada Tajrish Comprehensive Neurosurgical Center of Excellence, Shahid Beheshti University of Medical Sciences, Tehran, Iran

**Keywords:** COVID-19, mesenchymal stem cell, MAVS, S protein, SARS-CoV-2, mitochondria

## Abstract

The SARS-CoV-2, the virus that causes COVID-19, has infected millions of people worldwide. The symptoms of this disease are primarily due to pulmonary involvement, uncontrolled tissue inflammation, and inadequate immune response against the invader virus. Impaired interferon (IFN) production is one of the leading causes of the immune system’s inability to control the replication of the SARS-CoV-2. Mitochondria play an essential role in developing and maintaining innate cellular immunity and IFN production. Mitochondrial function is impaired during cellular stress, affecting cell bioenergy and innate immune responses. The mitochondrial antiviral-signaling protein (MAVS), located in the outer membrane of mitochondria, is one of the key elements in engaging the innate immune system and interferon production. Transferring healthy mitochondria to the damaged cells by mesenchymal stem cells (MSCs) is a proposed option for regenerative medicine and a viable treatment approach to many diseases. In addition to mitochondrial transport, these cells can regulate inflammation, repair the damaged tissue, and control the pathogenesis of COVID-19. The immune regulatory nature of MSCs dramatically reduces the probability of an immune rejection. In order to induce an appropriate immune response against the SARS-CoV-2, we hypothesize to donate mitochondria to the host cells of the virus. We consider MSCs as an appropriate biological carrier for mitochondria. Besides, enhancing the expression of MAVS protein in MSCs and promoting the expression of SARS-CoV-2 viral spike protein as a specific ligand for ACE2^+^ cells will improve IFN production and innate immune responses in a targeted manner.

## Introduction

The novel coronavirus disease 2019 (COVID-19), caused by severe acute respiratory syndrome coronavirus 2 (SARS-CoV-2), has caused a serious worldwide pandemic that has influenced different aspects of people’s lives. The disease is rapidly spreading, with almost 130 million confirmed cases and 2.8 million deaths by the end of March 2021 (WHO Coronavirus (Covid-19) Dashboard, 2021). RNA-based next-generation sequencing and proteomics approaches have revealed structural and non-structural proteins of the SARS-CoV-2. The S, N, M, and E genes encode structural proteins, while non-structural proteins are encoded by the open reading frame (ORF) regions (Huang et al., 2020). The virus enters the host cells by binding of its viral S protein to the angiotensin-converting enzyme 2 (ACE2) in different target organs, including the lung (type II surfactant-secreting alveolar cells), heart, kidney, brain, and intestine (Hoffmann et al., 2020). Initial manifestations of COVID-19 infection consist of fever, fatigue, myalgia, cough, dyspnea, and chest pain (Alimohamadi et al., 2020). Although most patients (85%) experience mild to moderate symptoms, some patients go through severe complications, including acute respiratory failure, acute respiratory distress syndrome (ARDS), septic shock, and acute cardiac injury (Dallan et al., 2020; Long et al., 2020). Despite the unclear underlying reasons for the life-threatening complications of COVID-19, severe inflammatory response due to high levels of pro-inflammatory mediators is theorized to be the leading cause of severe disease and death (Song et al., 2020). Following the identification and characterization of the SARS-CoV-2, a growing body of efforts has been made to suggest a curative approach for eliminating the complications of the disease. Considering the crucial role of the immune system in COVID-19 pathogenesis, targeting the cells and inflammatory mediators of the innate and adaptive immune system will provide interesting curative approaches to conquer the complications of the disease.

On the other hand, suggested therapeutic approaches should eliminate chronic and various complications of COVID-19, such as neurological, renal, cardiovascular, musculoskeletal, and long-term respiratory complications (Fraser, 2020; Kunutsor and Laukkanen, 2020; Heidarpour et al., 2021; Talasaz et al., 2021). Current treatments mainly consist of supportive care by invasive mechanical ventilation, antiviral drugs, and immunomodulatory agents (Rodríguez-Baño et al., 2021; Young et al., 2021). However, each of these therapies is only appropriate for a specific stage of the disease, and the effectiveness of these therapies is under question. Therefore, developing efficient therapies to control the COVID-19 complications is necessary.

## Supporting Evidence for Hypothesis

### Interferons Impairment in COVID-19

Following cell infection by SARS-CoV-2, many immune and non-immune cells are activated in response to the virus, which altogether leads to the release of considerable amounts of inflammatory cytokines such as IL-1, IL-6, and TNF-α, which is called “cytokine storm.” Ordinarily, it is expected that a well-coordinated immune response restricts the spread of the virus, whereas an excessive inflammatory response causes cytokine storm. It seems that this hyperinflammatory/immunodeficiency state is developed as a result of impaired innate immune response (Jamshidi et al., 2021). Type I interferons (IFNs), a group of innate immune cytokines, are secreted from virally infected cells and act on more than 7,000 genes that regulate critical cellular processes, including metabolism, survival, migration, and inhibition of virus replication and assembly (Wang and Fish, 2019). Many studies have proven the pivotal roles of IFNs in effective innate and adaptive immune responses against viral infections. In addition, the importance of IFNs in triggering antiviral immune responses during coronavirus infections is acknowledged (Lee and Ashkar, 2018; Banerjee et al., 2019).

It has been shown that severely impaired type I IFN response, marked as no IFN-β or low IFN-α, is the distinctive phenotype observed in critical COVID-19 patients. Impaired type I IFN response is associated with a higher viral load and uncontrolled inflammatory responses (Blanco-Melo et al., 2020; Hadjadj et al., 2020). In addition, some studies revealed that impaired IFN antiviral responses in the elderlies might emerge as a reason for poor prognosis in this group (Molony et al., 2018). Furthermore, the higher susceptibility of African-American populations to SARS-CoV-2 is attributed to the lower IFN-I production in response to the SARS-CoV-2 RNAs (Maiti, 2020).

In order to overcome impaired IFN-mediated immune response in COVID-19, previous studies proposed some solutions. For instance, it has been suggested to administer exogenous IFNs to improve the performance of the innate immune system. A retrospective cohort study in China revealed that early administration of inhaled aerosolized IFN-α2b results in lower death and shorter admission period. However, late administration of IFN-α2b is associated with the more extended admission period and higher mortality (Wang et al., 2020). In another study, forty-eight COVID-19 patients inhaled IFN-β1a, which led to a significant improvement on day 15 compared to the placebo group (Monk et al., 2021). Although administration of IFNs as an accepted therapeutic strategy improves immune responses, IFN therapy has faced controversial outcomes. It seems that the efficacy of administration of IFNs depends on the stage of the disease. In this line, using IFNs in the early stages generates desirable outcomes, whereas this strategy is ineffective in the severe or later stages of viral infection (Channappanavar et al., 2016).

As another solution, drug repurposing has been recently developed as an attractive topic due to lower costs and shorter timeline compared to standard drug discovery methods. Previously, it has been proposed that azithromycin, a macrolide antibiotic, can promote the response of type I IFN (IFN-β) and some virus recognition receptors in epithelial cells (Menzel et al., 2017; Li et al., 2019b). In this line, the antiviral efficacy of azithromycin on COVID-19 combined with hydroxychloroquine was showed *in vitro* (Damle et al., 2020). Trametinib, an anticancer drug, is another proposed drug against COVID-19, which acts via inhibition of mitogen-activated protein kinase (MEK) 1 and 2. It has been shown that MEK inhibitors triggered expression of some antiviral genes, including interferon regulatory factor 1 (IRF1), both at the mRNA and protein level, which results in improved IFN response (Lulli et al., 2017). However, drug repurposing is facing many challenges that may overweight its benefits. Serious adverse effects of some drugs, especially anticancer and immunomodulatory medications, lack of appropriate clinical trials and controversial reports regarding their effects are the main challenges.

It seems that targeting the IFN production pathway can improve the innate immune response, particularly in the early phases of COVID-19. It also prevents the pro-inflammatory overreactions or cytokine storm resulting from the rapid replication of SARS-CoV-2 in the absence of sufficient amounts of IFNs (Fung et al., 2020; Mozafari et al., 2020).

### Mitochondrial-Mediated Impairment of the Immune Response in COVID-19

Different cellular organelles participate in coordinating the IFN production and innate immune responses. Mitochondria take part in regulating cell bioenergy, cell metabolism, apoptosis, and reactive oxygen species (ROS) production and consumption. Besides, mitochondria play an essential role in inducing innate antiviral immune reactions mainly through stimulating IFN production. Studies have shown that following mitochondrial dysfunction, endothelial cells release large amounts of pro-inflammatory mediators such as IL-1, IL-6, and TNF-alpha and enhance the expression of intercellular adhesion molecule-1 (ICAM-1) that cause monocyte infiltration and activation (Choi S.J. et al., 2018). In order to trigger innate immune responses, the pathogen-associated molecular patterns (PAMPs) are identified by pattern recognition receptors (PRRs) such as intercellular retinoic acid-inducible gene-I-like receptors (RLRs). There are various RLRs, such as melanoma differentiation-associated protein 5 (MDA5) and retinoic acid-inducible gene I (RIG-1), that are the principal activators of mitochondrial antiviral pathways. These receptors are expressed in both immune and non-immune cells and activate several pathways, including interferon production, after recognizing single-strand and double-strand RNAs of viruses (Banoth and Cassel, 2018).

It has been shown that 26 out of 29 viral proteins of the SARS-CoV-2 interact with a significant number of mitochondrial proteins that participate in critical cellular metabolic pathways. This study suggests that the SARS-CoV-2 infection could result in mitochondrial dysfunction (Gordon et al., 2020). Besides, mitochondrial dysfunction is related to the clinical evidence that some metabolic disorders such as obesity or diabetes exponentially increase severe complications and mortality risk of COVID-19 (Bertsimas et al., 2020). It has been indicated that immune responses are affected by sex in which women display more effective innate immune responses than men. The data is along with a much higher mortality rate of COVID-19 in men. It can be attributed to the exclusive natural selection of healthy mitochondria in females. In this manner, mitochondria with malfunctioning or mutations that can be harmful to the females are eliminated (Iessi et al., 2021). Taken together, it seems that mitochondrial dysfunction plays a critical role in severe complications of COVID-19.

It has been hypothesized that mitochondrial-targeted ubiquinone (MitoQ), a mitochondrial-targeted antioxidant, could play a potential role in COVID-19 treatment. The particular MitoQ structure promotes its targeted accumulation within the mitochondria and sequestering the ROS (Ouyang and Gong, 2020). However, eliminating ROS only affects the narrow aspect of mitochondrial dysfunction while the impairment of mitochondrion-mediated immune response is still not resolved.

### MAVS as a Key Regulator of Innate Immune Response in COVID-19

Mitochondrial antiviral-signaling protein (MAVS), also known as IFN-β promoter stimulator I (IPS-1), is one of the essential messenger molecules for inducing the mitochondrial antiviral pathways, which was first described by Seth et al. in 2005 (Seth et al., 2005). MAVS consists of 540 amino acids and has three components, an N terminal caspase activation recruitment domain (CARD), a proline-rich domain, and a transmembrane C terminal domain (TM). Its gene is located on chromosome 20, and the protein itself is present in the outer membrane of the mitochondria (Wang et al., 2019). The binding of the virus antigens and RLRs complex to MAVS induces an innate immune response in the infected cells through two different mechanisms. The first mechanism is the activation of interferon regulatory factors (IRFs) and the nuclear factor kappa B (NF-κB) pathway that inhibits viral replication and assembly via enhancing the production of interferon-α and -β (Refolo et al., 2020). The second mechanism involves the activation of caspase-8 protein as a promoter of the internal pathway of apoptosis in the virus-infected cells that removes the infected cells and protects adjacent cells (El Maadidi et al., 2014).

The pivotal role of MAVS in IFN production makes it a valuable evading target for many viruses. In the coexistence of viruses and host cells, these pathogens have developed various strategies to conquer MAVS-mediated signaling. Cleavage of MAVS is one of the mechanisms involved in the dislocation of MAVS, resulting in the impairment of IFN-mediated immune response (Lu et al., 2020). For instance, it has been shown that different viruses such as cytomegalovirus (CMV), hepatitis C virus (HCV) and Zika virus can disrupt MAVS function and therefore impair IFN production (Choi H.J. et al., 2018; Ma et al., 2018; Yan et al., 2021). In this line, SARS-CoV-2 disrupts interferon response through damage to the MAVS protein, which leads to decreased expression of interferons in the early stages of infection and provides an excellent opportunity for the virus to multiply and spread (Lei et al., 2020). It is demonstrated that SARS-CoV-2 carries a protein gene called open reading frame-9b (ORF-9b) that inhibits the interferon response in the infected cells by inhibiting mitochondria and the MAVS signalosome. Additionally, it seems that disruption of mitochondrial energy production may induce lactic acid production and inhibit proper interferon response (Jiang et al., 2020). Besides, ORF-9b induces mitochondrial elongation by promoting ubiquitination and degradation of dynamin-like protein 1 (DNM1L) that participates in mitochondrial fission and maintenance (Shi et al., 2014).

It has been proposed to administer drugs that interact with ORFs and consequently prevent their role in virus replication and distribution. Regarding amino acid sequence searching, some chemical substances such as benzyl (2-oxopropyl) carbamate and 4-(dimethylamino) benzoic acid may prevent virus replication via targeting ORFs. However, they only inhibit a small range of viral proteins, and their efficacy in the treatment of COVID-19 has not been evaluated yet (Chen and Zhong, 2020).

### Mitochondrial Therapy and Mesenchymal Stem Cell

It is demonstrated that mitochondrial dysfunction plays a vital role in inhibiting antiviral response in the infected cells. It seems that restoring mitochondrial function and providing new healthy mitochondria could improve cell resistance against infection-related stress through regulating the bioenergy and innate immune response of the affected cells (Li et al., 2019a).

Some studies have suggested that mesenchymal stem cells (MSCs) are a viable option to transfer healthy mitochondria to infected cells. Mesenchymal stem cells are a group of non-hematopoietic stem cells that originate from a variety of adult tissues. Some of the characteristics of these cells, such as their ability to regenerate tissues, anti-inflammatory effects, immune-evading properties, and interaction with various intracellular or extracellular pathways, make them a new treatment for a variety of pathological conditions (Babajani et al., 2020). Mitochondrial transfer using the MSCs has been broadly evaluated in various organs, including cardiovascular, neurological, renal, and respiratory systems. MSCs have been utilized in ischemic vascular diseases to save human umbilical vein endothelial cells (HUVECs) with mitochondrial dysfunction. The mitochondrial donation recovered aerobic respiration and reduced apoptosis of ischemic endothelial cells (Liu et al., 2014). It is reported that mitochondria can move from the MSCs to neural cells and recover the bioenergetics and proliferation of the recipient cells (Babenko et al., 2018). It has been shown that mitochondrial transfer from MSCs to the respiratory epithelial cells of the asthma model can reduce epithelial cell apoptosis by reinstating mitochondrial function and regulating inflammation (Yao et al., 2018). In another study, transferring mitochondria to the stem cells improved oxidative phosphorylation and ATP production, resulting in increased proliferation, migration, and differentiation (Guo et al., 2020). Treatment of damaged renal cells with MSCs diminished ROS levels, downregulated mitochondrial apoptosis-related proteins and reduced cell apoptosis, shedding light on the beneficial effects of MSCs in regulating mitochondrial respiratory function (Geng et al., 2017). It was indicated that effective mitochondrial transfer is achieved by forming tunneling nanotubes (TNTs) between MSCs and the recipient cells, which allows direct intercellular communication (Jiang et al., 2016).

On the other hand, the possible benefits of MSCs administration in the treatment of COVID-19 have been widely studied in recent months (Rajarshi et al., 2020). MSCs reduce the complications of COVID-19 by affecting the pathological mechanisms of the disease. MSCs participate in immune regulation by modulating the cellular immunity and cytokine responses such as M1 to M2 macrophage alteration and activation of T reg cells (Zhang et al., 2019). They also enhance tissue repair in two different manners; first, by the paracrine effect on host cells of the tissue and second by differentiating to replace damaged cells (Li et al., 2017; Westhauser et al., 2017). Additionally, they regulate the function of the renin-angiotensin-aldosterone system (RAAS) by reducing angiotensin II accumulation in the alveoli. Angiotensin II plays an essential role in developing pulmonary fibrosis following the viral infection (Chen and Chou, 2018). MSCs increase the fluid clearance of alveoli and regulate the coagulation process, which reduces the chance of disseminated intravascular coagulation (DIC) and thrombosis (Wang et al., 2012).

## Hypothesis

**FIGURE 1:**
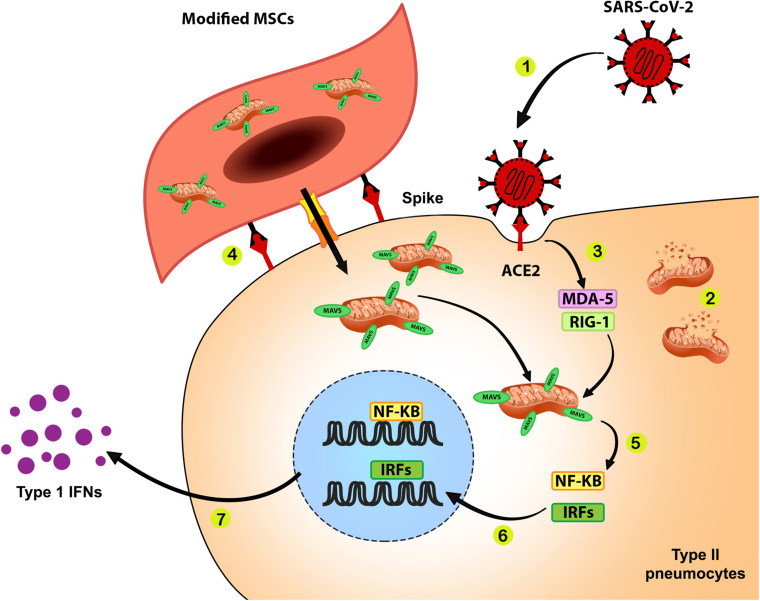
Improving anti-SARS-CoV-2 response during mitochondrial therapy by donating mitochondria with over-expressed MAVS. **1.** SARS-CoV-2 infects ACE2 expressing cells such as surfactant-secreting type II alveolar cells via interaction between the viral spike protein and ACE2. **2.** Cell infection with SARS-CoV-2 results in mitochondrial dysfunction and disturbances of bioenergetics and innate immune response in infected cells. **3.** The RNA content of SARS-CoV-2 is recognized by pattern recognition receptors such as MDA-5 and RIG-1. **4.** MSCs that express surface spike protein as ACE2 ligand and containing mitochondria with overexpressed MAVS transfer these modified mitochondria to the surfactant-secreting type II alveolar cells. **5.** Binding of activated MDA-5 and RIG-1 to the MAVS on the outer membrane of healthy donated mitochondria would activate NF-κB and IRF transcription factors. **6.** NF-κB and IRF translocate into the cell nucleus and upregulate genes related to the innate immune response against SARS-CoV-2. **7.** Function of NF-κB and IRF will lead to the production of IFNs, which play a pivotal role in the antiviral response.

**FIGURE 2:**
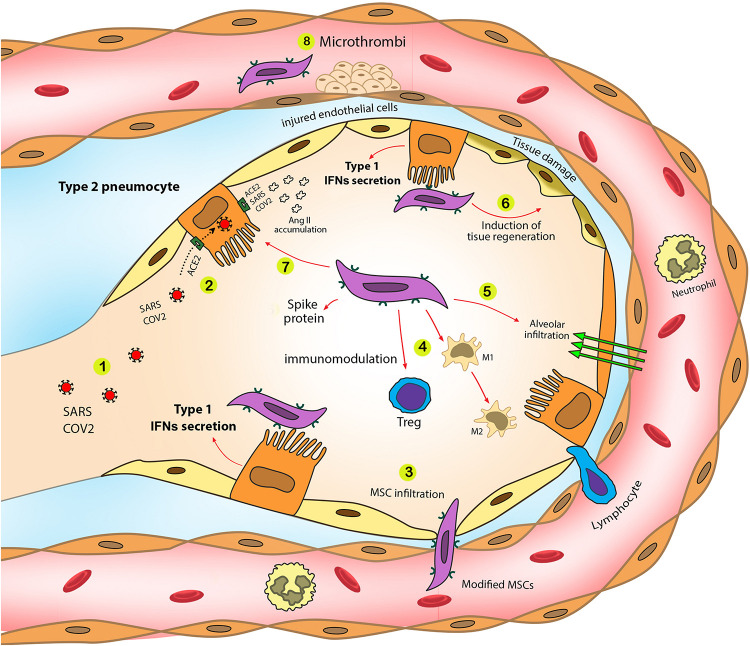
SARS-CoV-2 pathogenic effects and the role of modified MSCs in the eradication of SARS-CoV-2 complications. Modified MSCs trigger type 1 IFN production in a targeted manner. Additionally, utilizing MSCs as mitochondrial carrier eliminates various COVID-19 complications and synergically improve mitochondrial therapy in COVID-19. **1.** SARS-CoV-2 enters alveoli through airways. **2.** SARS-CoV-2 infects type II pneumocytes through the ACE2 receptor. **3.** Modified MSCs entrap in the lungs and infiltrate into the alveoli. **4.** MSCs induce immunomodulatory effects that control cytokine storm during severe stages of COVID-19. Modified MSCs increase Treg cell activity and M1 to M2 macrophage switch, diminishing inflammatory mediator production by M1 macrophages. **5.** Modified MSCs increase fluid clearance exudation from infected alveoli. **6.** Modified MSCs can participate in tissue regeneration to eliminate chronic fibrosis due to COVID-19. **7.** SARS-CoV-2 infection results in angiotensin II accumulation and following lung fibrosis. MSCs can modulate in angiotensin II production that reduces lung fibrosis. **8.** MSCs modulate coagulopathies during COVID-19.

We suggest that the *in vivo* transferring of mitochondria from manipulated MSCs that express viral S protein and have overexpressed MAVS protein could enhance the innate immune response and appropriate IFN production in a targeted manner. This hypothesis consists of three steps:

(1) It is possible to improve innate immune response by donating mitochondria with overexpressed MAVS protein as a crucial initiator of the immune response of the cell against the virus. Enhancing MAVS expression can compensate for the destructive effects of viral proteins such as ORF-9b on immune-related proteins of mitochondria, which results in IFN production in its due time. The appropriate action of mitochondria produces sufficient amounts of IFNs as a crucial innate immunity arm that inhibits replication and assembly of new viruses. It also eliminates damaged and infected cells by promoting apoptosis that limits infection. The proposed roles of donated mitochondria with over-expressed MAVS to the type II pneumocytes are shown in Figure 1.

(2) It is possible to utilize MSCs as a biological carrier for mitochondria to donate these organelles to the infected and damaged cells in COVID-19 cases. The immunomodulatory functions of MSCs enable us to transport considerable amounts of mitochondria to the damaged organs in an immune reaction-free condition. In addition to the carrier role of MSCs, these cells can provide extra benefits due to their inherent characteristics, such as immunomodulatory effect by controlling cytokine release, regulating RAAS function, increasing alveolar fluid clearance, and decreasing the chance of hypercoagulation (Jamshidi et al., 2021). The roles of MSCs in eliminating COVID-19 complications are shown in Figure 2.

(3) In order to make the donation process more targeted, we can use ACE2 specific ligands to target susceptible cells for infection. Viral S protein seems to be the best option for administration as a specific ACE2 ligand. The SARS-CoV-2 S protein is preserved among all human coronaviruses and participates in the virus recognition, attachment, and entry into the susceptible cells (Huang et al., 2020). Using S protein enables us to target all possible SARS-CoV-2 hosts (ACE2^+^ cells) in various parts of the body, such as the lungs, oral mucosa, GI tract, kidney, and heart (Xu et al., 2020). Besides, SARS-CoV-2 S protein induces antibody production that brings about vaccine-like effects. It has been shown that the administration of viral S protein can provoke an immune response and produce anti-spike IgG (Keech et al., 2020). In another study, injection of manipulated MSCs, which expressed surface viral S proteins, into an animal model showed an effective antibody production (Liu et al., 2020).

## Evaluation of the Hypothesis

This hypothesis can be evaluated by conducting *in vitro* and *in vivo* experimental studies to address the hypothesis’s building block questions.

### *In vitro* Studies

The viral vector containing the SARS-CoV-2 S protein gene will be transduced to MSCs using lentivirus transduction based on previous protocols (Meyerrose et al., 2008). Utilizing lentivirus vectors enables MSCs to produce S protein for a long period which raises the chance of targeted therapy of susceptible cells. The transduction success rate should be measured by GFP-labeled vectors. Administration of 3-(4,5-dimethylthiazol-2-yl)-2,5-diphenyl-2H-tetrazolium bromide (MTT) assay is recommended to evaluate MSCs viability after cell manipulation. Spike protein expression on the surface of modified MSCs can be studied by using specific antibodies against the spike protein and the flow cytometry method.

In order to enhance IFN production, the spike protein-expressing MSCs can be transfected with MAVS gene-containing plasmid by electroporation protocol based on previous protocols (Peister et al., 2004). The transfection success rate could be measured by GFP-labeled vectors, and MSCs viability after the manipulation should be assessed by MTT assay. Western blotting on cell lysates would evaluate MAVS expression and successful translation after plasmid transfection. Determining the subcellular localization of MAVS and accurate translocation of produced MAVS to the cell mitochondria is critical in this study. It has been shown that MAVS is localized in the mitochondrial outer membrane, and MAVS’s mislocalization abolishes its activity (Seth et al., 2005). Evaluation of MAVS protein translocation to the mitochondrial outer membrane should be done by utilizing immunofluorescence assay and fluorescent-labeled antibodies for MAVS protein and mitochondrial outer membrane described by previous studies (Vazquez et al., 2017).

It is expected that MSCs transfer their mitochondria to the ACE2^+^ cells. Thus, mitochondrial transfer from modified MSCs to the infected surfactant-secreting type II alveolar cells should be evaluated using fluorescence labeling of mitochondria and FACS method after *in vitro* coculture of modified MSCs and SARS-Cov-2 infected type II pneumocytes. It is expected that mitochondrial transfer to the infected cells results in four changes: (1) reduction of oxidative stress, (2) enhancement of IFN production, (3) decreasing the apoptosis rate in the initial stages of SARS-CoV-2 infection, and (4) reduction of viral load in infected surfactant-secreting type II alveolar cells. IFN production can be measured by using the ELISA method. To assess the effects of mitochondrial transfer and improved IFN production on apoptosis of type II pneumocytes, annexin V/PI staining and flow cytometry is recommended. Viral replication in type II pneumocytes is expected to be interrupted after the intervention. Therefore, viral load should be measured by quantitative reverse transcription-polymerase chain reaction (qRT-PCR).

### *In vivo* Studies

*In vivo* studies could include evaluating spike^+^ MSCs distribution within the animal body, histopathological changes of the target organs of SARS-CoV-2, and measuring anti-SARS-CoV-2 IgM and IgG after the intervention. The animal model should be susceptible to SARS-CoV-2 infection with a sensitized respiratory tract. It is possible to transduce the model with adenovirus or adeno-associated virus that expresses human ACE2 protein (Muñoz-Fontela et al., 2020).

In order to assess spike^+^ MSCs distribution in the body of the COVID-19 animal model, MSCs will be labeled with ultrasmall superparamagnetic iron-oxide nanoparticles (USPIO), and these cells could be tracked using MRI until 21 days after injection to the COVID-19 animal model (Crabbe et al., 2010). Histopathological changes of the lungs should be examined for inflammatory cell infiltration and fibrin deposition in the lung tissue of the COVID-19 animal model in treated and untreated groups at days 0, 2, 7, 10, 14, and 21 after the treatment by H&E staining and light microscopy. These changes should be compared with the untreated COVID-19 animal model. Anti-spike protein antibody titers would also be compared in treated and untreated groups. For this purpose, 20 days after the treatment, the blood samples will be obtained from both groups, and anti-spike antibody titers will be measured using ELISA.

## Discussion and Future Direction

Most complications of COVID-19 come from the impaired innate and adaptive immune responses against SARS-CoV-2. This immunodeficiency/hyperinflammation state results in high viral load and cytokine storm. Considering the crucial role of mitochondria in inducing innate and adaptive immune responses by promoting IFN production and activating antiviral pathways, we suggested that transferring healthy mitochondria will improve innate immune response and prevent the distribution of the infection in the body. Besides, healthy mitochondria will improve cellular bioenergy that is impaired during metabolic syndrome and infection. In order to boost mitochondrial-mediated immune response, we suggest increasing the expression of mitochondrial antiviral-signaling protein (MAVS) protein as a pivotal player of the cellular innate immune response.

Targeted therapy of COVID-19 provides several benefits, including targeted mitochondria delivery to eligible cells, reduced side effects of direct mitochondria application, and a lesser number of administered mitochondria needed. It has been shown that the administration of mitochondria without any carrier causes inflammation. Considering the bacterial origin of mitochondria, the application of uncovered mitochondrial DNA, which is similar to bacterial DNA, induces toll-like receptor-9 (TLR9)-mediated inflammatory responses (Oka et al., 2012). Thus, designing appropriate carriers with the ability of targeted therapy improves the outcome. MSCs have been administered for targeted therapy in different pathological situations. These cells are immune evasive, which results in reducing the chance of immune rejection (Babajani et al., 2020). Additionally, MSCs and their exosomes possess innate therapeutic effects in different conditions, including cancer and COVID-19 (Golchin et al., 2020). The safety of clinical administration of MSCs was approved by many clinical studies (Thompson et al., 2020), and a growing body of studies are applying MSCs from different sources for the treatment of COVID-19 (available on www.clinicaltrials.gov). Additionally, it seems that expressing S protein, a ligand for ACE2, on the surface of MSCs will make the delivery of mitochondria more targeted. Besides, viral S protein might promote antibody production that induces vaccine-like effects (Kuate et al., 2007). Taken together, the anti-inflammatory features of spike expressing MSCs, along with their ability to eliminate complications of COVID-19, could make them valuable mitochondrial-targeted carriers for prevention and therapy in COVID-19.

Utilizing non-cellular or cellular carriers may provide some advantages and disadvantages. However, selecting a semi-biologic carrier that offers both cellular and non-cellular cargos benefits is the optimal aim in targeted mitochondrial therapy. As a miniature copy of the original cell, exosomes possess various beneficial functions of MSCs, including immunomodulation and regeneration (Jafari et al., 2021). Exosomes can be found in the cellular secretions that regulate several intercellular communications. Intercellular transfer of exosome-mediated mitochondria is reported in some studies (Maremanda et al., 2019). It has been reported that exosomes with encapsulated mitochondria can transfer these organelles from myeloid-derived regulatory cells (MDRCs) to CD4^+^ T cells that resulted in alteration of pro-inflammatory function, bioenergetic, T cell differentiation and signaling of mitochondria in chronic inflammatory diseases (Hough et al., 2018). Administration of exosomes would provide some advantages over cells, including circumventing invasive cell harvesting, controlling dosage and potency, supply storable and easy-accessible sources, and extra stability (Yin et al., 2019). Thus, exosomes can be the future options for mitochondrial transfer-based therapies.

To the best of our knowledge, no studies have focused on administering MAVS overexpressed mitochondrial therapy to treat COVID-19. Therefore, it is essential to investigate the potential therapeutic effects of mitochondrial therapy for COVID-19.

## Data Availability Statement

The original contributions presented in the study are included in the article/supplementary material, further inquiries can be directed to the corresponding author/s.

## Author Contributions

AB, PH-M, SA, and HN: conceptualization. AB, PH-M, and SA: investigation. AB, PH-M, and EJ: writing—original draft preparation. AB, PH-M, EJ, and HN: writing—review and editing. HN: supervision. All authors read and agreed to the submitted version of the manuscript.

## Conflict of Interest

The authors declare that the research was conducted in the absence of any commercial or financial relationships that could be construed as a potential conflict of interest.
